# Impact of Overhead Irrigation Timing on Ornamental Plant Phytotoxicity Following Preemergence Herbicide Applications

**DOI:** 10.3390/plants14111710

**Published:** 2025-06-04

**Authors:** Chengyao Yin, Christopher Marble, Jianjun Chen, Adam Dale

**Affiliations:** 1 Mid-Florida Research and Education Center, Institute of Food and Agricultural Sciences, University of Florida, Apopka, FL 32703, USA; chengyao.yin@ufl.edu (C.Y.); jjchen@ufl.edu (J.C.); 2 Entomology and Nematology Department, Institute of Food and Agricultural Sciences, University of Florida, Gainesville, FL 32611, USA; agdale@ufl.edu

**Keywords:** container, herbicide, irrigation, nursery, ornamental plants, phytotoxicity

## Abstract

The use of preemergence herbicides is the primary method of controlling weeds in container-grown ornamental plants, but it may cause injury to common popular ornamentals. The objective of this research was to evaluate the use of overhead irrigation to reduce phytotoxicity in ornamental plants. Dimethenamid-P and flumioxazin were applied at standard label rates to container-grown coneflower (*Echinacea purpurea*), lady fern (*Anthyrium filix-femina*), and blue plumbago (*Plumbago auriculata*). Plants were subjected to one of four irrigation regimes at the time of herbicide treatment, including receiving 1.3 cm of overhead irrigation before treatment, immediately after treatment, both immediately before and after treatment, and no irrigation until the next irrigation cycle resumed at 4 h after treatment. For all three species, irrigation timing had minimal effect on visual injury ratings following treatment with dimethenamid-P, as injury was minimal overall. Severe injury was observed following treatment with flumioxazin, but significant recovery was noted in both lady ferns and echinacea when irrigation was applied immediately after treatment. The results indicate that irrigating plants immediately after treatment could improve crop tolerance to preemergence herbicide applications and should be further investigated as an injury management strategy for container-grown ornamental plants.

## 1. Introduction

Weeds can significantly reduce the growth of containerized nursery crops through competition [1] and can decrease the salability of container-grown plants, as customers typically demand weed-free plants [2]. Over time, weeding technology has gradually developed from manual hand weeding to chemical methods using herbicides. Although many growers realize the benefits of integrating a diversity of weed management approaches [3,4], the use of preemergence herbicides is the predominate method of weed management in container nurseries, primarily due to a lack of postemergence options and crop safety concerns, paired with the high labor costs associated with hand weeding [2,5,6,7,8].

The application of preemergence herbicides, however, is often associated with phytotoxicity injury on ornamentals. While commercially available preemergence herbicides have been tested and shown to be safe on many ornamentals [9], not all ornamental taxa can be tested, and many popular species, such as *Hydrangea* spp., tropical plants, ferns, ornamental grasses, and herbaceous perennials, are susceptible to herbicide injury, restricting options from a growers’ perspective. While environmental factors such as temperature, rainfall, humidity, and soil type can have a significant impact on phytotoxicity [10,11], damage to certain ornamentals is difficult to avoid unless focusing solely on non-chemical approaches. Phytotoxicity is also very difficult to predict as ornamental plants comprise a vast number of taxa. Previous studies have shown significant differences in response to preemergence herbicide applications from herbaceous plants, woody ornamentals, and ornamental grasses, even when testing herbicides within the same chemical class [12,13]. An additional concern is the aesthetic appearance of ornamental plants. As they are produced for their ornamental value and marketed and sold based on aesthetic attraction, even minor levels of injury cannot be tolerated, which is in contrast to other crops valued by their yield [14]. Economic implications resulting from phytotoxicity to ornamentals have not been thoroughly investigated, likely due to the many variables at hand. Outcomes from phytotoxicity could result in a total loss, increased production time, or accepting lower than market value for a particular crop in some instances. Total crop failures with no salvageable plants could potentially result in losses of over USD 100,000 per acre, depending upon the species, not including lost materials and labor costs. Prolonging production times can also disrupt operations, future sales forecasts, and potting scheduling, resulting in lost customers or contracts. Due to these negative implications, reducing phytotoxicity, or even the chances of phytotoxicity, is very important from an economic standpoint.

Past research has focused on several methods to mitigate herbicide phytotoxicity in various cropping systems. In field crops, particularly corn, rice, sorghum, and wheat, herbicide safeners are commonly employed to reduce herbicide phytotoxicity [15]. Safeners are organic compounds that essentially make herbicides less active or inactive on crop plants and active through different mechanisms, typically enhancing metabolic detoxification of herbicides in crop plants without reducing efficacy on key weed species [15]. While these products are viable in monoculture systems, they have limitations, as they are typically very crop-specific and their efficacy can be influenced by many factors, including temperature, soil moisture, soil structure, and the rate of application of the safener [16]. As nursery crops are produced in vastly different environments and thousands of taxa are produced, safeners are not currently considered a viable option for most nursery growers.

One practice that can potentially reduce injury from herbicides is overhead irrigation timing. For ornamental plant producers, irrigation is necessary for ideal crop growth and also plays a crucial role in improving herbicide performance, as most preemergence herbicides require irrigation to be activated [17,18]. In other cropping systems, irrigation has been shown to mitigate injury from certain herbicides. In one previous study [19], injury to bermudagrass (*Cynodon dactylon*) was significantly reduced following applications of topramezone, carfentrazone, and multiple tank mixtures when irrigation (0.7 cm) was applied immediately after herbicide treatment compared with bermudagrass that was not irrigated following herbicide treatment. Similar findings were also reported by Brewer et al. [20] with regard to improvements in turfgrass quality following immediate irrigation. However, these authors also reported decreased herbicide efficacy in goosegrass (*Eleusine indica*), the target weed species, although this would not be of concern with preemergence herbicides as they need to be watered in soon after application and are soil-active, not foliar-active [9].

Irrigation timing following preemergence herbicide applications has not been extensively evaluated in nursery crops, but some previous work exists. Richardson et al. [21] reported significantly lower injury to Camellia (*Camellia japonica*) and azalea (*Rhododendron* spp.) following applications of diuron when overhead irrigation was applied within 1 h of herbicide application. In ornamental plant production, there is often little to no tolerance for any phytotoxicity from agrochemicals. If post-treatment irrigation could reduce injury from certain preemergence herbicides that may cause only minor injury, growers may have many more herbicide options at their disposal if tools like post-treatment irrigation could be fine-tuned [22]. Therefore, the objective of this research was to determine the influence of pre- and/or post-herbicide treatment irrigation timings on the injury and growth of three commonly produced and herbicide-sensitive ornamental plant species.

## 2. Results

**Figure 1:**
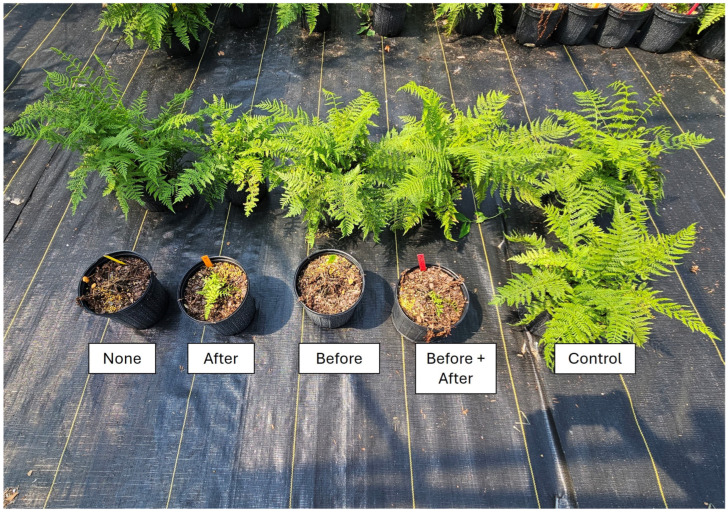
Lady fern (*Anthyrium filix-femina*) at 2 weeks following an over-the-top application of dimethenamid-P (top row) or flumioxazin (bottom row) at standard label rates. Following treatment, plants were subjected to one of five irrigation timings (equivalent to 1.3 cm of irrigation via a watering can), including (1) Before = irrigation applied only immediately before herbicide application; (2) After = irrigation applied only immediately after herbicide treatment; (3) Before + After = irrigation applied both immediately before and immediately after herbicide treatment; (4) None = no irrigation was applied until 4 h following herbicide treatment, when regular irrigation scheduling was resumed. The non-treated control group received no herbicide application and was irrigated at 4 h during the normal irrigation.

*Anthyrium filix-femina:* Minimal injury was noted in all plants treated with dimethenamid-P, but at 1 WAT, plants irrigated immediately after treatment or before and after treatment had injury ratings similar to the non-treated control (Table 1). Injury decreased in all treatments at 2 and 4 WATs, with the only plants having significantly higher injury than the non-treated control being plants that received no irrigation immediately before or after treatment (none). By 8 WATs, plants had fully recovered, with no observable injury or significant growth reduction compared to plants that did not receive a herbicide treatment (Figure 1).

**Figure 2:**
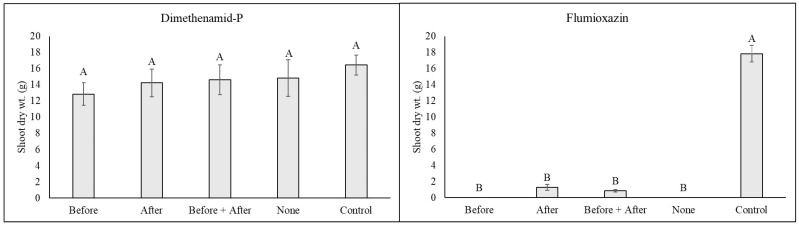
Lady fern (*Anthyrium filix-femina*) shoot dry weight at 8 weeks following an over-the-top application of dimethenamid-P or flumioxazin at standard label rates. Following treatment, plants were subjected to one of five irrigation timings (equivalent to 1.3 cm of irrigation via a watering can), including (1) Before = irrigation applied only immediately before herbicide application; (2) After = irrigation applied only immediately after herbicide treatment; (3) Before + After = irrigation applied both immediately before and immediately after herbicide treatment; (4) None = no irrigation was applied until 4 h following herbicide treatment, when regular irrigation scheduling was resumed. The non-treated control group received no herbicide application and was irrigated at 4 h during the normal irrigation scheduling. Means and standard errors are shown and are pooled over experiments conducted in 2023 and 2024. Means followed by the same letter are not significantly different according to Tukey’s HSD test (0.05).

In contrast to dimethenamid-P, plants treated with flumioxazin were severely injured regardless of irrigation timing treatment, with injury ratings ranging from 9.7 to 10.0 at 1 WAT (Table 1). Similar observations were noted at 2 WATs, but by 4 WATs, plants that received irrigation immediately after herbicide treatment began to recover (Figure 2). In conclusion, plants irrigated immediately after treatment had almost fully recovered, with mean injury ratings of only 1.3. Significant recovery was also observed in plants irrigated both before and after treatment (mean ratings of 4.3 ± SE). However, plants that received no irrigation immediately after herbicides were applied, including plants only irrigated before herbicide treatment or no irrigation before or after treatment, did not survive. While recovery was noted in both treatments in which irrigation was applied after treatment, significant growth reductions were observed in all treatments (Figure 2).

**Figure 3:**
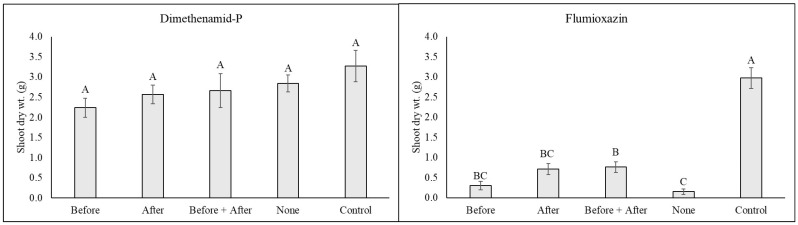
Purple coneflower (*Echinacea purpurea*) shoot dry weight at 8 WATs following an over-the-top treatment with dimethenamid-P or flumioxazin at standard label rates. Following treatment application, plants were subjected to one of five irrigation timings (equivalent to 1.3 cm of irrigation via a watering can), including (1) Before = irrigation applied only immediately before herbicide application; (2) After = irrigation applied only immediately after herbicide treatment; (3) Before + After = irrigation applied both immediately before and immediately after herbicide treatment; (4) None = no irrigation was applied until 4 h following herbicide treatment, when regular irrigation scheduling was resumed. The non-treated control group received no herbicide application and was irrigated at 4 h during the normal irrigation scheduling. Means and standard errors are shown and are pooled over experiments conducted in 2023 and 2024. Means followed by the same letter are not significantly different according to Tukey’s HSD test (0.05).

*Echinacea purpurea*. All plants showed signs of transplant stress at 1 WAT, characterized by some leaf loss and general chlorosis, resulting in controls having some “injury” symptoms (Table 2). However, trends were still detectable after treatment application. At 1 WAT, the highest injury in dimethenamid-P-treated plants was found for those that were either not irrigated following treatment or only irrigated before treatment. By 2 WATs, the only plants with injury ratings similar to non-treated plants were those irrigated both before and after herbicide treatment. By 4 WATs, no differences were observable in any treatment, and all plants were similar in quality to the non-treated control group throughout the remainder of the experiment. Similarly, no differences in growth index were observed between any of the dimethenamid-P-treated plants, regardless of irrigation timing (Figure 3).

**Figure 4:**
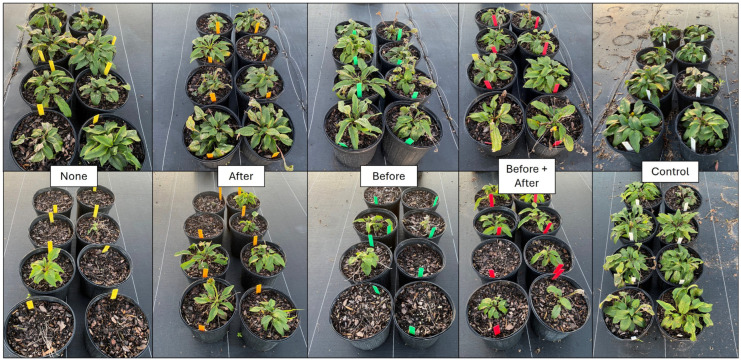
Purple coneflower (*Echinacea purpurea*) at 4 weeks following an over-the-top treatment with dimethenamid-P (top row) or flumioxazin (bottom row) at standard label rates. Following treatment application, plants were subjected to one of five irrigation timings (equivalent to 1.3 cm of irrigation via a watering can), including (1) Before = irrigation applied only immediately before herbicide application; (2) After = irrigation applied only immediately after herbicide treatment; (3) Before + After = irrigation applied both immediately before and immediately after herbicide treatment; (4) None = no irrigation was applied until 4 h following herbicide treatment, when regular irrigation scheduling was resumed. The non-treated control group received no herbicide application and was irrigated at 4 h during the normal irrigation scheduling.

All Echinaceae plants treated with flumioxazin showed a high level of injury (Figure 4), but plants irrigated after herbicide treatment or both before and after herbicide treatment showed significantly higher recovery, as noted by injury ratings at both 2 and 4 WATs (Table 3). By 8 WATs, the lowest injury ratings were observed in plants irrigated after treatment (3.4) and both before and after treatment (2.3), which had significantly lower injury than plants that were only irrigated before treatment (mean of 6.1 ± SE) or received no irrigation until the normal irrigation schedule was resumed (mean rating of 7.8 ± SE). Significant growth reductions were observed in all flumioxazin-treated plants compared with non-treated plants (Figure 3). However, the shoot dry weight was highest in plants irrigated after or both before and after flumioxazin treatment.

**Figure 5:**
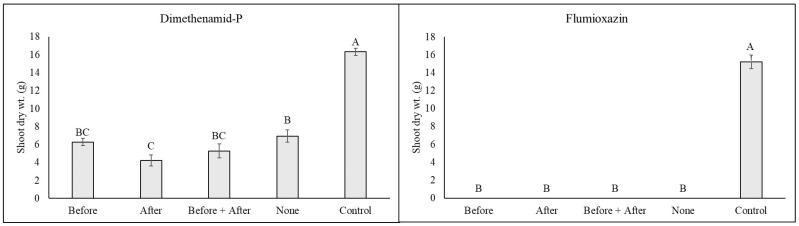
Plumbago (*Plumbago auriculata*) shoot dry weight at 8 WATs following an over-the-top treatment with dimethenamid-P or flumioxazin at standard label rates. Following treatment application, plants were subjected to one of five irrigation timings (equivalent to 1.3 cm of irrigation via a watering can), including (1) Before = irrigation applied only immediately before herbicide application; (2) After = irrigation applied only immediately after herbicide treatment; (3) Before + After = irrigation applied both immediately before and immediately after herbicide treatment, and (4) None = no irrigation was applied until 4 h following herbicide treatment, when regular irrigation scheduling was resumed. The non-treated control group received no herbicide application and was irrigated at 4 h during the normal irrigation scheduling. Means and standard errors are shown and are pooled over experiments conducted in 2023 and 2024. Means followed by the same letter are not significantly different according to Tukey’s HSD test (0.05).

**Figure 6:**
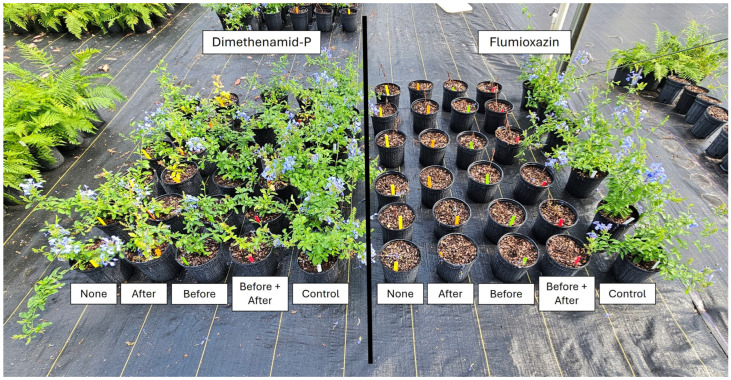
Plumbago (*Plumbago auriculata*) at 4 WATs following an over-the-top treatment with dimethenamid-P (left) or flumioxazin (right) at standard label rates. Following treatment application, plants were subjected to one of five irrigation timings (equivalent to 1.3 cm of irrigation via a watering can), including (1) Before = irrigation applied only immediately before herbicide application; (2) After = irrigation applied only immediately after herbicide treatment; (3) Before + After = irrigation applied both immediately before and immediately after herbicide treatment; (4) None = no irrigation was applied until 4 h following herbicide treatment, when regular irrigation scheduling was resumed. The non-treated control group received no herbicide application and was irrigated at 4 h during the normal irrigation scheduling.

*Plumbago auriculata.* At 1 WAT, injury from dimethenamid-P was minimal in all treatments, with the highest injury rating observed in plants that received no irrigation before or after herbicide treatment (Table 3). At 2 WATs, all plants had injury ratings similar to non-treated plants, except plants that were irrigated immediately after treatment, which was in contrast to the results observed with other species. This trend continued through 8 WATs, and while some injury was noted, all plants would have been considered marketable regardless of irrigation timing. While plants were generally marketable and appeared healthy with minor herbicide injury, significant growth reductions were observed in the shoot dry weight with all herbicide treatments, resulting in significant reductions in plant growth compared with the non-treated control group, regardless of irrigation timing (Figure 5). Regardless of irrigation timing, all plants treated with flumioxazin died by 1 WAT, with mean injury ratings of 10 in all treatments, and no recovery was observed (Figure 6).

## 3. Discussion

While injury was minimal on plants treated with dimethenamid-P in all three ornamental species, injury tended to be highest when irrigation was delayed for 4 h following herbicide application, especially compared to both treatments in which irrigation was immediately applied following treatment. Dimethenamid-P is a chloroacetamide herbicide and inhibits lipid biosynthesis, leading to growth disruption and plant death. It is labeled for use on several hundred ornamental plants, but injury can sometimes be observed on new growth, often from the oil-based emulsifiable concentrate formulation, which is exacerbated in high temperatures [9]. Irrigating immediately after treatment decreased injury by approximately 10% to 30% in lady fern and purple coneflower at early evaluation dates compared to plants that were only irrigated before treatment or when irrigation was delayed for 4 h (the “none” treatment). Irrigation to wet plant foliage before herbicide treatment had little to no effect on phytotoxicity, as injury ratings were similar between plants that were irrigated only before treatment and those that were not irrigated before or immediately after treatment. Although injury was affected by irrigation timing, plant growth was largely unaffected, as plants tended to recover and grow normally after 2 to 4 weeks following application with dimethenamid-P.

As expected, plant injury following treatment with flumioxazin was greater than dimethenamid-P, regardless of irrigation timing. This is probably due to the flumioxazin mode of action, which inhibits protoporphyrinogen oxidase, a key enzyme for chlorophyll biosynthesis, disrupting chlorophyll production and causing rapid plant damage and death. Flumioxazin is formulated as a soluble concentrate (SureGuard^®^), is only registered for use as a directed application to the soil or potting media, and is not labeled for over-the-top use on ornamental species other than certain conifers [23]. Regardless of irrigation timing, an over-the-top application of flumioxazin resulted in complete plant death to all plumbago, but irrigation immediately following herbicide application significantly improved safety in both lady fern and purple coneflower. Within the plant, flumioxazin causes rapid desiccation and necrosis of plant tissues as the protoporphyrinogen oxidase enzyme is inhibited. This leads to the accumulation of protoporphyrin, which oxidizes to protoporphyrin IX and, in the presence of sunlight, interacts with ground-state oxygen to form singlet oxygen that essentially oxidizes lipids and proteins, causing a loss of chlorophyll and rapid cell wall degradation [24]. Immediate irrigation likely limited foliar absorption and led to increased safety in lady fern and purple coneflower. Similar to dimethenamid-P, irrigating immediately before treatment had no effect on plant survivability, injury, or growth.

To our knowledge, no previous research has investigated the effects of irrigation timing on herbicide injury to mitigate phytotoxicity caused by dimethenamid-P or flumioxazin in container nursery crops. Richardson et al. [21] previously evaluated increasing plant safety following applications of Dichlobenil, but very little formal testing has been conducted to date. As improvements were noted in this study for both dimethenamid-P and flumioxazin when irrigation was applied immediately after treatment, further research is warranted to determine if injury can be mitigated from other herbicides following application to different ornamental species.

While irrigating immediately after treatment has been shown to significantly reduce injury in other planting systems, such as turfgrasses [19], reductions in postemergence herbicide efficacy have also been reported [20], as postemergence herbicides typically need to dry on plant foliage to be translocated effectively [25]. In contrast, preemergence herbicides must be irrigated following application to be activated, with most products typically requiring 1.3 cm within 3 to 7 days for consistent control [9]. Others have also shown improved efficacy of preemergence herbicides when irrigation is applied within 2 days after herbicide application compared to longer time intervals [26]. A separate study found that no differences in efficacy were observed (100% weed control through 8 WAT), which was expected, both due to the short nature of the study and the fact that differing irrigation times of only 4 h in a nursery setting would be considered inconsequential due to the typical frequency of irrigation in this type of production system [27]. Overall, this study indicates that, while results differed slightly between species and herbicides, injury decreased with immediate irrigation after treatment. As these herbicides must be irrigated soon after treatment, regardless of phytotoxicity concerns, it would be recommended to apply preemergence herbicides and then irrigate as soon as possible after the application is concluded. This would provide two primary benefits, including reducing the chances of phytotoxicity by washing foliar-active herbicides off of plant shoots while also activating the herbicide and potentially improving weed control. More research is needed on how crop canopy structure and size affect the herbicide distribution within the container, but this method could potentially result in more herbicide reaching the target area of the media surface on plants with dense foliage, as it could move herbicide off the shoots and onto the media surface. Future research should focus on utilizing timely irrigation to improve the safety of preemergence herbicides when applied over the top of notoriously sensitive ornamental species such as *Hydrangea*, tropical plants, succulents, and other plants in which few herbicide options exist.

## 4. Materials and Methods

All experiments were conducted in the fall of 2023 and spring of 2024 at the Mid-Florida Research and Education Center in Apopka, FL, USA. Three ornamental plant species were selected for evaluation, including purple coneflower (*Echinacea purpurea*), blue plumbago (*Plumbago auriculata*), and lady fern (*Anthyrium filix-femina*). All three species were selected based on their sensitivity to preemergence herbicides and documented injury following herbicide applications [9,22,28] For each species, 5 cm liners were transplanted into trade gallon (3.0 L) containers filled with a pinebark:sand (90:10 v:v) substrate amended with a controlled release fertilizer [Osmocote^®^ Plus micronutrients 21-4-8 N-P-K (8–9 mo)], (ICL Specialty Fertilizers, Dublin, OH, USA) at 4.7 kg m^−3^ via incorporation prior to potting.

Following potting, purple coneflower pots were placed on a full sun nursery pad and received overhead irrigation daily via two irrigation cycles totaling 1.3 cm per day, whereas plumbago and lady fern were placed in a shaded greenhouse (40% of ambient light) and received 0.7 cm of irrigation daily via overhead irrigation. Approximately one week after potting, two herbicides, including dimethenamid-P (Tower^®^ 6.0 EC, BASF Corp., Research Triangle Park, NC, USA) and flumioxazin (SureGuard^®^ SC, Nufarm Inc., Alsip, IL, USA), were applied over the top of plants at standard label rates (1.7 and 0.4 kg ai ha^−1^ for dimethenamid-P and flumioxazin, respectively) using a CO_2_ backpack sprayer calibrated to deliver 468 l ha^−1^ application volume using a flat fan nozzle (8008 flat fan nozzle, TeeJet Technologies, Wheaton, IL, USA). Dimethenamid-P was chosen for evaluation as it is a common “over the top” preemergence herbicide used in container nursery production, and although no safety data have been established in lady fern or blue plumbago, injury has been observed with purple coneflower previously [29]. Flumioxazin was chosen as it can be highly injurious to numerous ornamental species when applied over the top of foliage [9]. The objective of the study was to determine if timely overhead irrigation can mitigate injury from moderately (dimethenamid-P) or significantly (flumioxazin) injurious preemergence herbicides applied over top of sensitive ornamental plants.

Irrigation treatments (timings) consisted of plants that were irrigated either immediately before herbicide treatment, immediately after herbicide treatment, both immediately before and immediately after herbicide treatment, or received no irrigation (before or after) for 4 h following herbicide treatment, when normal irrigation scheduling resumed. Irrigation treatments (timings) were simulated by using watering cans that were filled with a predetermined volume of water to deliver 1.3 cm of overhead irrigation to all plants in an individual treatment group. A non-treated control group was included for each ornamental species and for each herbicide evaluated. Each experiment was repeated in time, with the second experimental run commencing upon the completion of the first experimental run.

Following herbicide and irrigation treatments, plants were grouped by species in a completely randomized design with 8 single pot replications for each herbicide and irrigation treatment. Data collected included visual injury ratings on a 0 to 10 scale, where 0 = no injury; 1 = very minor injury; 2 = minor injury; 3 = maximum acceptable injury; 4 = moderate injury; 5 = half the plant exhibiting injury; 6 = significant injury; 7 = very significant injury; 7 = severe injury; 8 = very severe injury; 9 = almost complete plant death but some green or living tissues observed; and 10 = dead plant or no living tissues observed at 1, 2, 4, and 8 weeks after treatment (WAT). At trial conclusion (8 WAT), shoot dry weight was determined on all plants by clipping shoots at the soil line and drying shoots in a forced air oven until constant weight was reached. All data were analyzed using JMP 16.0 software (SAS Institute, Cary, NC, USA), and prior to analysis, normality was confirmed using quantile plots. All data were inspected for normality using a normal quantile and Shapiro–Wilk test. Injury ratings were arcsine-transformed when needed to meet the assumptions of normality but were back-transformed for presentation. Following ANOVA analysis, post hoc mean separation was carried out when ANOVA detected an effect of treatment, using Tukey’s Honest Significant Difference test (*p* = 0.05) to make all possible comparisons between treatments. As results did not differ over experimental runs, data were pooled for both experimental replications. As the objective of the study was to determine the effect of irrigation timing on herbicide phytotoxicity to different ornamental species and not to directly compare herbicides or ornamental species tolerance, each herbicide was analyzed separately for each ornamental species, and separate control groups were included for each herbicide. Weed control was also assessed by taking percent coverage ratings on a 0 to 100% scale, where 0 = no weed coverage and 100 = 100% weed coverage, accounting for all weed species present. However, weed growth was non-existent in all herbicide-treated pots and minimal in non-treated pots; thus, data were not analyzed or reported.

## Figures and Tables

**Table 1:** *Anthyrium filix-femina* (Lady fern) mean injury ratings (±standard error) following pre- and post-application irrigation after over-the-top treatment with dimethenamid-P and flumioxazin herbicides. Results are pooled over two experimental runs conducted in 2023 and 2024.

	Dimethenamid-P				Flumioxazin			
	1 WAT ^z^	2 WATs	4 WATs	8 WATs	1 WAT	2 WATs	4 WATs	8 WATs
Irrigation timing ^y^	Injury ratings (0 to 10) ^x^							
Before treatment	1.5 (0.4) a ^w^	0.3 (0.2) ab	0.0 (0.0) b	0.0 (0.0) a	10.0 (0.0) a	10.0 (0.0) a	10.0 (0.0) a	10.0 (0.0) a
After treatment	0.8 (0.2) ab	0.3 (0.2) ab	0.1 (0.1) b	0.0 (0.0) a	9.7 (0.2) a	9.5 (0.3) a	8.2 (0.5) b	1.3 (0.9) bc
Before + After treatment	0.3 (0.2) b	0.0 (0.0) b	0.0 (0.0) b	0.0 (0.0) a	9.9 (0.1) a	10.0 (0.0) a	9.3 (0.3) a	4.3 (1.3) b
None	1.8 (0.4) a	1.7 (0.7) a	0.5 (0.2) a	0.0 (0.0) a	9.8 (0.1) a	10.0 (0.0) a	10.0 (0.0) a	10. (0.0) 0 a
Non-treated control	0.0 (0.0) b	0.0 (0.0) b	0.0 (0.0) b	0.0 (0.0) a	0.0 (0.0) b	0.0 (0.0) b	0.0 (0.0) c	0.0 (0.0) c

^z^ WATs = weeks after herbicide and irrigation treatments were applied. ^y^ Before treatment = plants irrigated before herbicide treatments were applied; After treatment = plants irrigated immediately after herbicide treatments were applied; Before + After = plants that were irrigated immediately before and after herbicide treatments were applied; None = plants that were irrigated at 4 h following herbicide treatment; Non-treated control = plants that were not treated with herbicide and irrigated at 4 h following herbicide treatment. ^x^ Injury ratings were taken on a 0 to 10 scale, where 0 = no plant injury and 10 = dead plant (no visible green shoot tissue). ^w^ Means within a column followed by the same letter are not significantly different according to Tukey’s HSD test (0.05).

**Table 2:** *Echinacea purpurea* (purple coneflower) mean injury ratings (±standard error) following pre- and post-application irrigation after over-the-top treatment with dimethenamid-P and flumioxazin herbicides. Results are pooled over two experimental runs conducted in 2023 and 2024.

	Dimethenamid-P				Flumioxazin			
	1 WAT ^z^	2 WATs	4 WATs	8 WATs	1 WAT	2 WATs	4 WATs	8 WATs
Irrigation timing ^y^	Injury ratings (0 to 10) ^x^							
Before treatment	2.6 (0.2) ab ^w^	1.7 (0.2) a	1.8 (0.3) a	1.9 (0.2) a	8.1 (0.4) a	9.1 (0.4) ab	9.8 (0.3) a	6.1 (1.1) ab
After treatment	1.8 (0.3) b	1.7 (0.2) a	1.9 (0.3) a	1.7 (0.2) a	3.1 (0.4) b	7.3 (0.5) c	6.7 (0.9) b	3.4 (0.9) bc
Before + After treatment	1.6 (0.3) b	1.4 (0.2) ab	1.8 (0.2) a	1.5 (0.1) a	7.3 (0.4) ab	7.8 (0.6) bc	3.8 (0.9) c	2.3 (0.8) c
None	2.9 (0.2) a	1.7 (0.2) a	1.9 (0.2) a	1.6 (0.2) a	8.0 (0.4) a	9.6 (0.2) a	9.5 (0.5) a	7.8 (0.9) a
Non-treated control	1.6 (0.3) b	0.9 (0.2) b	1.3 (0.2) a	1.4 (0.2) a	1.1 (0.3) c	0.8 (0.1) d	1.4 (0.2) c	1.5 (0.2) c

^z^ WATs = weeks after herbicide and irrigation treatments were applied. ^y^ Before treatment = plants irrigated before herbicide treatments were applied; After treatment = plants irrigated immediately after herbicide treatments were applied; Before + After = plants that were irrigated immediately before and after herbicide treatments were applied; None = plants that were irrigated at 4 h following herbicide treatment; Non-treated control = plants that were not treated with herbicide and irrigated at 4 h following herbicide treatment. ^x^ Injury ratings were taken on a 0 to 10 scale, where 0 = no plant injury and 10 = dead plant (no visible green shoot tissue). ^w^ Means within a column followed by the same letter are not significantly different according to Tukey’s HSD test (0.05).

**Table 3:** *Plumbago auriculata* (blue plumbago) mean injury ratings (±standard error) following pre- and post-application irrigation after over-the-top treatment with dimethenamid-P and flumioxazin herbicides. Results are pooled over two experimental runs conducted in 2023 and 2024.

	Dimethenamid-P				Flumioxazin			
	1 WAT ^z^	2 WATs	4 WATs	8 WATs	1 WAT	2 WATs	4 WATs	8 WATs
Irrigation timing ^y^	Injury ratings (0 to 10) ^x^							
Before treatment	0.6 (0.2) ab ^w^	0.0 (0.0) b	0.3 (0.2) ab	0.0 (0.0) b	10.0 (0.0) a	10.0 (0.0) a	10.0 (0.0) a	10.0 (0.0) a
After treatment	0.6 (0.2) ab	0.9 (0.3) a	0.3 (0.2) ab	0.0 (0.0) b	10.0 (0.0) a	10.0 (0.0) a	10.0 (0.0) a	10.0 (0.0) a
Before + After treatment	0.8 (0.3) a	0.0 (0.0) b	0.0 (0.0) b	0.0 (0.0) b	10.0 (0.0) a	10.0 (0.0) a	10.0 (0.0) a	10.0 (0.0) a
None	1.2 (0.4) a	0.1 (0.1) b	0.8 (0.1) a	1.2 (0.4) a	10.0 (0.0) a	10.0 (0.0) a	10.0 (0.0) a	10.0 (0.0) a
Non-treated control	0.0 (0.0) b	0.0 (0.0) b	0.0 (0.0) b	0.0 (0.0) b	0.0 (0.0) b	0.0 (0.0) b	0.0 (0.0) b	0.0 (0.0) b

^z^ WATs = weeks after herbicide and irrigation treatments were applied. ^y^ Before treatment = plants irrigated before herbicide treatments were applied; After treatment = plants irrigated immediately after herbicide treatments were applied; Before + After = plants that were irrigated immediately before and after herbicide treatments were applied; None = plants that were irrigated at 4 h following herbicide treatment; Non-treated control = plants that were not treated with herbicide and irrigated at 4 h following herbicide treatment. ^x^ Injury ratings were taken on a 0 to 10 scale, where 0 = no plant injury and 10 = dead plant (no visible green shoot tissue). ^w^ Means within a column followed by the same letter are not significantly different according to Tukey’s HSD test (0.05).

## Data Availability

The raw data supporting the conclusions of this article will be made available by the authors on request.

## References

[1] Fretz T.A. (1973). Herbicide-impregnated mulches for weed control in container nursery stock. Sci. Hort..

[2] Naruhn G.P., Peteinatos G.G., Butz A.F., Möller K., Gerhards R. (2021). Efficacy of various mechanical weeding methods—Single and in combination—In terms of different field conditions and weed densities. Agronomy.

[3] Deng X. (2022). Current advances in the action mechanisms of safeners. Agronomy.

[4] Yu P., Marble S.C. (2022). Practice in nursery weed control—Review and meta-analysis. Front. Plant Sci..

[5] Case L.T., Mathers H.M., Senesac A.F. (2005). A review of weed control practices in container nurseries. HortTechnology.

[6] Judge C.A., Neal J.C. (2006). Preemergence and early postemergence control of selected container nursery weeds with Broadstar, OH2, and Snapshot TG. J. Environ. Hort..

[7] Chandel A.K., Tewari V.K., Kumar S.P., Nare B., Agarwal A. (2018). On-the-go position sensing and controller predicated contact-type weed eradicator. Curr. Sci..

[8] Jiang W., Quan L., Wei G., Chang C., Geng T. (2023). A conceptual evaluation of a weed control method with post-damage application of herbicides: A composite intelligent intra-row weeding robot. Soil Tillage Res..

[9] Neal J.C., Chong J.H., Williams-Woodward J., Springer M.T. (2017). 2017 Southeastern US Pest Control Guide for Nursery Crops and Landscape Plantings.

[10] Negrisoli E., Velini E.D., Tofoli G.R., Cavenaghi A.L., Martins D., Morelli J.L., Costa A.G.F. (2024). Selectivity of pre-emergence herbicides to sugarcane treated with nematicides. Planta Daninha.

[11] Oliveira R.S., Constantin J., Hernandes A.I.F.M., Inoue H.M., Marchiori O., Ramires A.C. (2001). Tolerance of five cassava (*Manihot esculenta*) cultivars to herbicides. Planta Daninha.

[12] Derr J.F., Salibu S. (1996). Preemergence herbicide effects on nursery crop root and shoot growth. J. Environ. Hort..

[13] Staats D., Klett J.E. (1993). Evaluation of weed control and phytotoxicity of preemergence herbicides applied to container-grown herbaceous and woody plants. J. Environ. Hort..

[14] Hamouz P., Hamouzová K., Novotná K. (2015). Effects of spring herbicide treatments on winter wheat growth and grain yield. Sci. Agric. Bohem..

[15] Hatzios K.K. (1991). An overview of the mechanisms of action of herbicide safeners. Z. Naturforsch..

[16] Abu-Qare A.W., Duncan H.J. (2002). Herbicide safeners: Uses, limitations, metabolism, and mechanisms of action. Chemosphere.

[17] Kelley L. (2012). Irrigation to Improve Herbicide Performance.

[18] Zollinger R., Bernards M., Young B., Peterson D., Kruger G. (2018). Efficacy of water-conditioning adjuvants for dicamba-tolerant soybean. Pesticide Formulation and Delivery Systems: 38th Volume, Innovative Application, Formulation, and Adjuvant Technologies.

[19] Kerr R.A., McCarty L.B., Brown P.J., Harris J., McElroy J.S. (2019). Immediate irrigation improves turfgrass safety to postemergence herbicides. HortScience.

[20] Brewer J.R., Craft J.C., Askew S.D. (2022). Influence of posttreatment irrigation timings and herbicide placement on bermudagrass and goosegrass (*Eleusine indica*) response to low-dose topramezone and metribuzin programs. Weed Sci..

[21] Richardson B.M., Gilliam C.H., Wehtje G.R., Fain G.B. (2006). Postemergence oxalis control with diuron: Minimizing crop injury with timely irrigation. J. Environ. Hort..

[22] Karimmojeni H., Rezaei M., Tseng T.M., Mastinu A. (2022). Effects of metribuzin herbicide on some morpho-physiological characteristics of two Echinacea species. Horticulturae.

[23] (2017). Sureguard^®^ SC Herbicide Product Label.

[24] Senseman S.A. (2007). Herbicide Handbook.

[25] Roggenbuck F.C., Penner D., Burow R.F., Thomas B. (1993). Study of the enhancement of herbicide activity and rainfastness by an organosilicone adjuvant utilizing radio labelled herbicide and adjuvant. Pestic. Sci..

[26] Adjesiwor A.T., Bhattarai P., Montgomery C., Gomm J. (2024). Weed control efficacy and dry bean response to preemergence herbicide incorporation timing. Agronomy.

[27] Grant O.M., Davies M.J., Longbottom H., Atkinson C.J. (2009). Irrigation scheduling and irrigation systems: Optimizing irrigation efficiency for container ornamental shrubs. Irrig. Sci..

[28] Dupont Y.L., Strandberg B., Damgaard C. (2018). Effects of herbicide and nitrogen fertilizer on non-target plant reproduction and indirect effects on pollination in *Tanacetum vulgare* (Asteraceae). Agric. Ecosyst. Environ..

[29] (2017). Tower^®^ Herbicide Product Label.

